# NETest2.0^®^ Demonstrates Superior Monitoring Performance Compared with Chromogranin A in Neuroendocrine Tumor Surveillance

**DOI:** 10.3390/cancers18142206

**Published:** 2026-07-09

**Authors:** Kiarash Mashayekhi, Mark Kidd, Anthony Gulati

**Affiliations:** 1 Department of Surgery, University of North Dakota School of Medicine & Health Sciences, Grand Forks, ND 58203, USA; kia.mashayek@alumni.usc.edu; 2 Wren Laboratories, Branford, CT 06405, USA; 3 Department of Medical Oncology, Stamford Health, Stamford, CT 06902, USA; agulati@stamhealth.org

**Keywords:** neuroendocrine tumors, NETest2.0^®^, chromogranin A, liquid biopsy, monitoring, recurrence, progression

## Abstract

**Background/Objectives**: Reliable biomarkers for longitudinal surveillance of neuroendocrine tumors (NETs) remain an unmet clinical need. Chromogranin A (CgA), the most widely used circulating biomarker, is limited by low sensitivity, substantial biologic variability, and poor concordance with radiologic progression. NETest2.0^®^ is a blood-based multigene transcriptomic liquid biopsy designed to dynamically assess NET biologic activity. This study compared serial NETest2.0^®^ measurements with CgA for monitoring disease progression in a real-world registry cohort. **Methods**: Patients with histologically confirmed NETs enrolled in the RegisterNET program (NCT02270567) who had paired blood samples and contemporaneous clinical assessment were included. NETest2.0^®^ scores were derived from quantitative RT-PCR analysis of a 51-gene transcript panel and expressed on a 0–100 scale. Serum CgA levels were measured using standard clinical immunoassays. Imaging-based disease assessment was performed using CT, MRI, and/or ^68^Ga-somatostatin receptor PET/CT with RECIST 1.1 criteria applied where appropriate. Longitudinal percentage changes (Δ) between sequential measurements were evaluated using predefined NETest2.0^®^ thresholds and compared with the conventional CgA threshold (>50%). NETest2.0 Δ thresholds of >0% and >5% were evaluated a priori: >0% as a high-sensitivity threshold capturing any upward transcriptomic drift, and >5% as a more conservative threshold intended to reduce minor biological or analytical fluctuation. Receiver operating characteristic (ROC) analysis, operating characteristics, multivariable analysis (MVA), and logistic regression analysis (LRA) were performed. **Results**: A total of 191 patients were analyzed. Exploratory ROC analysis demonstrated superior discrimination for progression using serial NETest2.0^®^ changes compared with changes in CgA (AUC: 0.893 vs. 0.538; *p* < 0.0001). In the primary surveillance analysis, NETest2.0^®^ thresholds of >0% and >5% achieved AUCs of 0.860 (95% CI: 0.803–0.906) and 0.822 (95% CI: 0.760–0.873), respectively, both significantly superior to CgA (AUC: 0.553, 95% CI: 0.480–0.625; both *p* < 0.0001). NETest2.0^®^ >0% demonstrated the highest sensitivity (86.4%), whereas NETest2.0^®^ >5% achieved the optimal balance of sensitivity (70.5%), specificity (93.9%), and overall accuracy (88.5%). In multivariable and logistic regression analyses, changes in NETest2.0^®^ were the strongest independent predictor of progression (all *p* < 0.0001; odds ratios: 52.99–61.26), whereas CgA did not significantly contribute to progression prediction. **Conclusions**: Serial NETest2.0^®^ assessment significantly outperformed CgA for monitoring NET disease activity and progression. These findings support the integration of NETest2.0^®^ into molecularly informed surveillance strategies to complement imaging and improve longitudinal monitoring of patients with NETs.

## Simple Summary

Reliable biomarkers for longitudinal surveillance of neuroendocrine tumors (NETs) remain an unmet clinical need. Chromogranin A (CgA), the most widely used circulating biomarker, has limited sensitivity and poor concordance with radiologic progression. This study compared serial NETest2.0^®^ measurements with CgA for monitoring NET progression in 191 patients enrolled in RegisterNET (NCT02270567). NETest2.0^®^ scores were generated using a 51-gene RT-PCR transcriptomic panel. Serial changes were analyzed using predefined NETest2.0^®^ (>0%, >5%) and CgA (>50%) thresholds. NETest2.0^®^ significantly outperformed CgA for progression detection (AUC 0.860–0.893 vs. 0.538–0.553; *p* < 0.0001). NETest2.0^®^ >5% achieved 88.5% accuracy, supporting its role as a superior molecular surveillance biomarker for NET monitoring.

## 1. Introduction

Although previously considered rare, gastroenteropancreatic neuroendocrine neoplasms (GEP-NEN) are now recognized as relatively common malignancies, with an incidence approaching 3.6 per 100,000 and a prevalence of approximately 35 per 100,000 [1,2]. Their clinical importance is magnified by the fact that 50–70% of patients present with metastatic disease and many require prolonged surveillance because recurrence may occur years after apparently curative therapy [3].

Neuroendocrine tumors (NETs) are biologically heterogeneous and may arise from multiple anatomic sites, including the gastrointestinal tract, pancreas, lung, and less commonly unknown primary locations [4,5]. Although GEP-NEN represents the largest clinically studied subgroup, the need for reliable longitudinal biomarkers extends across NET subtypes because patients frequently require prolonged surveillance, treatment monitoring, and assessment for delayed progression [6,7]. NETest2.0 was developed to measure a neuroendocrine tumor-associated transcriptomic signature rather than a site-specific secretory product, providing a biological rationale for evaluating the assay across diverse NET populations [8].

Current monitoring strategies rely principally on cross-sectional or functional imaging [9] and on blood biomarkers such as Chromogranin A (CgA). Imaging is indispensable but has recognized limitations in the setting of microscopic residual disease, early recurrence, and frequent longitudinal monitoring [10]. Repeated imaging also imposes cost, patient burden, and, in the case of CT-based surveillance, cumulative radiation exposure [11,12].

CgA is the best-known circulating biomarker in neuroendocrine tumor (NET) practice. However, its clinical utility is constrained by poor sensitivity, substantial intra- and inter-individual variability, non-standardized assay platforms, and multiple false-positive causes including proton-pump inhibitor therapy, renal dysfunction, and inflammatory states [13,14,15]. Importantly, CgA often correlates poorly with tumor burden [3,16] or treatment response [14], and guidelines have moved away from using this assay as it lacks the sensitivity/specificity needed for precision monitoring [17,18]. This leaves a major unmet need for more biologically informative biomarkers to aid with clinical decision-making.

NETest2.0^®^ is a blood-based, machine-learning-enhanced multigene transcript assay that measures a 51-gene neuroendocrine-specific expression signature in whole blood [8]. The assay is designed as a liquid biopsy, capturing circulating tumor-associated transcripts and generating a standardized 0–100 score with a validated positivity threshold of 50. By measuring dynamic transcriptomic changes over time rather than a single secretory protein, NETest2.0^®^ may better reflect dynamic disease-associated biologic activity [8,19].

The present study was designed to compare serial NETest2.0^®^ measurements with CgA for monitoring NET disease activity in a real-world registry population. An earlier study identified serial changes in NETest2.0^®^ were effective as monitors of progression [19]. We hypothesized that changes in NETest2.0^®^ between sequential blood samples would provide more accurate discrimination of stable versus progressive disease than changes in CgA.

## 2. Materials and Methods

**Figure 1:**
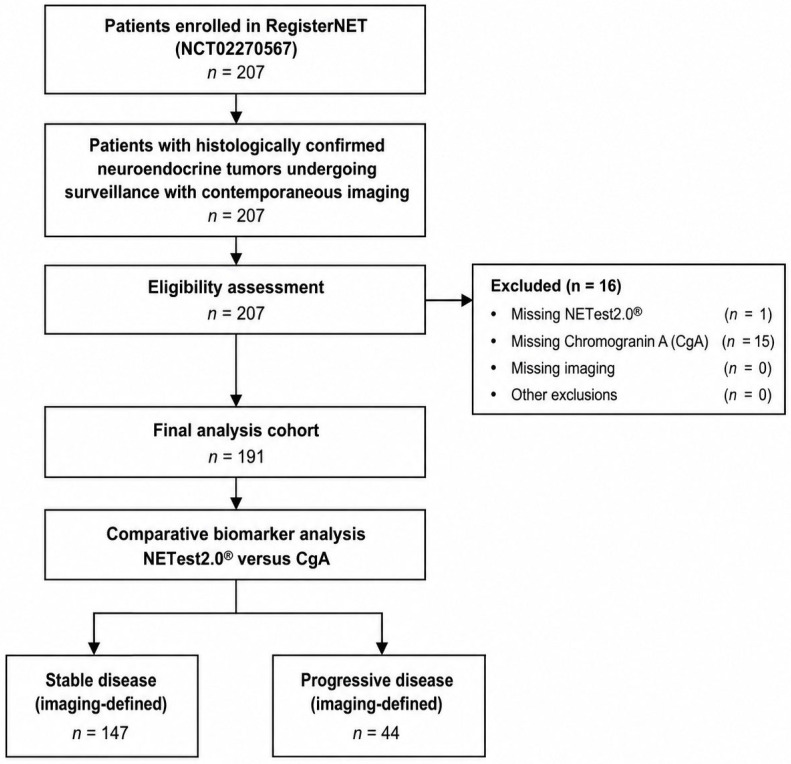
STROBE participant flow diagram. Patients were identified from the RegisterNET observational registry (ClinicalTrials.gov identifier NCT02270567). A total of 207 patients with histologically confirmed neuroendocrine tumors undergoing surveillance with contemporaneous imaging assessment were evaluated for inclusion. Sixteen patients were excluded because of incomplete biomarker data: one missing NETest2.0^®^ result and 15 missing Chromogranin A (CgA) results. No patients were excluded because of missing imaging or other protocol-defined criteria. The final analysis cohort consisted of 191 patients, including 147 with imaging-defined stable disease and 44 with imaging-defined progressive disease, who were included in the comparative evaluation of NETest2.0^®^ and CgA.

Study design and oversight: This registry-based observational study used prospectively collected biospecimens and predefined clinical follow-up within routine care pathways in the RegisterNET program (ClinicalTrials.gov NCT02270567) [20]. The objective was to evaluate the clinical validity and comparative surveillance performance of NETest2.0^®^ across diverse NET management settings, including postoperative follow-up, treatment monitoring, and routine longitudinal assessment. The study was conducted and reported in accordance with the Strengthening the Reporting of Observational Studies in Epidemiology (STROBE) guidelines (Figure 1). A completed STROBE checklist is provided as Supplementary Table S1.

Ethics approval was obtained from the Western Institutional Review Board/WCG Board (20150174) [20]. Samples and associated clinical information were de-identified before analysis, and all clinical, demographic, treatment, and imaging data were entered into a central study database.

Patients and clinical assessment: Patients (*n* = 191) with histologically confirmed neuroendocrine tumors were eligible if paired blood samples and corresponding clinical assessments were available. Tumor staging and grading were according to the European Neuroendocrine Tumor Society (ENETS) tumor–node–metastasis (TNM) staging and grading system [21]. Surgical and non-surgical treatment was according to NET guidelines and under consideration of individual patients’ condition. Regular physical examination, blood tests and morphologic imaging were undertaken every 3–6 months, and functional imaging every 6–12 months or anytime earlier if suspicious findings on morphologic imaging were evident.

For this study, two blood samples were collected during routine care (Table 1). Imaging was performed concurrently with the second blood draw. Progression was defined by RECIST 1.1 where measurable disease permitted formal assessment. When RECIST measurement was not applicable, clinically meaningful progression was defined by unequivocal radiologic progression of non-measurable disease, new lesions, progression on functional imaging, or physician-adjudicated progression requiring change in management. Imaging interpretation and clinical disease status assignment were performed as part of routine care and were not based on NETest2.0 results. Imaging modality selection was independent of biomarker results and was performed according to physician judgment and institutional practice. Whenever feasible, the same imaging modality was used longitudinally in a given patient.

Sample collection and molecular analysis: Peripheral whole blood (2 mL) was collected into tubes containing 4 mL of proprietary guanidinium hydrochloride-based RNA stabilization buffer [22]. Because messenger RNA was extracted from whole blood, no additional pre-analytical processing was required. RNA isolation, cDNA synthesis, quantitative PCR, and computational processing were performed as previously described for NETest2.0^®^ [8,19].

NETest2.0^®^ results were expressed as a continuous score from 0 to 100 and dichotomized using the validated positivity threshold of ≥50. Serum CgA was measured using standard clinical immunoassays performed according to local institutional laboratory practice. CgA assay platforms were not standardized across sites, reflecting real-world usage and measurement of this test.

**Figure 2:**
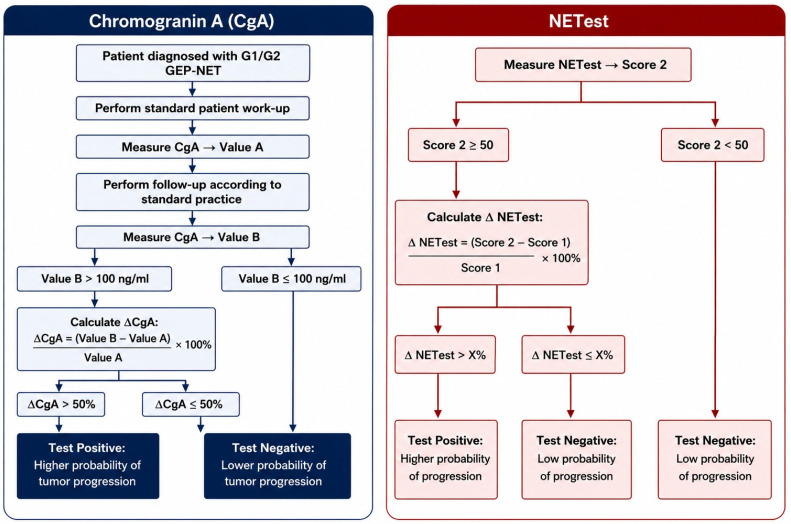
Paradigm used to evaluate changes for Chromogranin A (CgA) per the CASPAR study [23] and NETest score for outcome. We evaluated the following Δ thresholds (change between 2nd and 1st score): >0% and >+5% for NETest.

For longitudinal monitoring, percentage change between the second and first measurement (Δ) was calculated. NETest2.0^®^ delta thresholds of >0% and >5% were assessed. The >0% threshold was selected to test whether any increase in transcriptomic activity between sequential samples could identify progression with high sensitivity. The >5% threshold was selected as a more conservative operating point intended to reduce the influence of minor biological or analytical variation and improve specificity. These thresholds were evaluated against the conventional CgA Δ > 50% threshold used in the CASPAR surveillance framework [23]. The approach is described in Figure 2.

NETest2.0 uses a locked computational algorithm that converts normalized expression values from 51 NET-associated transcripts into a standardized 0–100 activity score. The algorithm was developed and validated in prior NET cohorts and was not retrained or modified for the present analysis [8]. The current study evaluated the longitudinal clinical performance of the locked assay output. The analytical precision of NETest2.0 has been previously established using replicate clinical specimens, operators, instruments, and assay runs [24]. Intra-assay and inter-assay variability were within predefined analytical acceptance criteria, supporting the use of serial score changes for longitudinal monitoring. The >5% threshold was included to provide a conservative operating point above minimal analytical and biological fluctuation.

Statistical analysis: Data were presented as positive/negative, median scores (range) and mean ± SD. Diagnostic discrimination for progressive versus non-progressive disease was assessed using continuous variables (NETest2.0 scores 0–100; or CgA [ng/mL] with receiver operating characteristic (ROC) analysis). Areas under the ROC curve (AUCs) were presented with standard errors and 95% confidence intervals. A 3-point AUC ROC was also generated to examine the clinical performance of the different thresholds. Pairwise AUC comparisons were interpreted using the statistical outputs provided in the source analysis (DeLong-type comparisons). Sensitivity, specificity, positive likelihood ratio (PLR), negative likelihood ratio (NLR), positive predictive value (PPV), negative predictive value (NPV), and overall accuracy were summarized for CgA and each NETest2.0^®^ threshold. Multivariable analyses (MVA) and logistic regression analyses (LRA) were undertaken using the different thresholds (T > 0% or T > 5%) and included age, gender, grade, lymph node, liver metastases and CgAT > 50%. Sensitivity analyses included Pearson and Spearman correlations; bootstrap confidence intervals (5000 iterations); exclusion of observations beyond the 95th percentile of PFS and/or |ΔNETest|; winsorization at the 1st and 99th percentiles; PFS quartile analysis; median PFS split analysis; logistic regression for progression status; and leave-one-out influence analysis. Progression status was coded 0 = stable and 1 = progressive.

All analyses were performed using Prism version 9.4 for Windows (GraphPad Software Inc, La Jolla, CA, USA, www.graphpad.com) and MedCalc Statistical Software v23.2.1 (MedCalc Software Ltd., Ostend, Belgium; http://www.medcalc.org; 2017). Statistical significance was defined as *p* < 0.05.

## 3. Results

### 3.1. Study Cohort and Monitoring Framework

A total of 191 patients contributed paired blood samples and paired clinical assessments. The median (range) age was 60 years (19–88 years) with a gender ratio of 109:82 (Male:Female). One hundred (52.5%) were G1, 79 (41.4%) were G2 and 12 (6.1%) were G3. One hundred and thirty (68.1%) were lymph node positive; and 84 (44%) had liver disease.

### 3.2. Sample Analyses

All analyses were performed on matched sampling intervals with contemporaneous imaging at the second timepoint. The median timepoint between samples was 6 months (range: 1–12 months). The registry cohort was intentionally pragmatic and reflected routine surveillance practice across multiple clinical scenarios. This design was intended to reflect real-world surveillance practice rather than protocolized imaging conditions. Although this approach introduces variability, it also increases the generalizability of the findings across routine NET surveillance settings. Imaging modalities and timing were not protocol-standardized and may have introduced heterogeneity in progression classification. Although this approach introduces variability, it also increased the generalizability of the findings across routine NET surveillance settings. Disease status at follow-up was categorized as stable/non-progressive or progressive on the basis of imaging and clinical status adjudication by NET specialists [19].

The analytic framework first examined whether change in NETest2.0^®^ between sequential samples provided similar or better discrimination than change in CgA. Exploratory ROC analysis compared changes in NETest2.0^®^ scores with changes in CgA levels directly, after which, a primary monitoring analysis evaluated predefined NETest2.0^®^ thresholds against the conventional CgA threshold used in the CASPAR framework [23]. Threshold analyses were subsequently performed to evaluate clinically interpretable operating points for longitudinal surveillance.

### 3.3. Exploratory ROC Analysis

**Figure 3:**
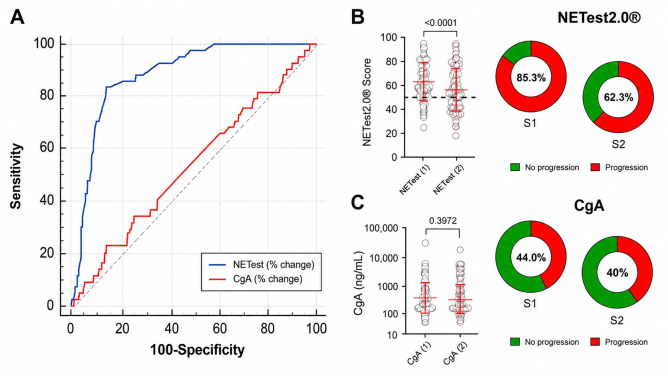
AUC for changes in results between the two sample times. (**A**) NETest2.0^®^ demonstrated substantially greater discrimination for progression than CgA (AUC 0.893 vs. 0.538; *p* < 0.0001). (**B**) Distribution of NETest2.0^®^ results for the first and second sampling times. The percentage positive is included. (**C**) Distribution of CgA results for the first and second sampling times. The percentage positive is included. Mean ± SD. Dotted black line is upper limit of normal for NETest2.0^®^.

In the exploratory analysis, changes in NETest2.0^®^ scores ([Sample 2 − Sample 1]/Sample 1) showed a significant discrimination for progressive disease, with an AUC of 0.893 ± 0.025 (95% CI: 0.84–0.933) (Figure 3A). Scores were significantly altered between the first and second samples (*p* < 0.0001, Figure 3B(left)). Scores were positive in 85.3% (sample 1) and in 62.3% of the second samples (Figure 3B(right)). The reduction in NETest2.0 positivity between the first and second samples occurred predominantly in patients classified as stable/non-progressive and is consistent with the expected behavior of a dynamic activity biomarker in a surveillance cohort that includes treated patients and patients with biologically quiescent disease.

By contrast, changes in paired CgA samples showed near-chance performance, with an AUC of 0.538 ± 0.0503 (95% CI: 0.465–0.610). Unlike NETest scores, CgA levels were not significantly altered between the first and second samples (*p* = 0.40, Figure 3C(left)). In addition, levels were positive in only 44.0% (Sample 1) and in 40% of the second samples (Figure 3C(right)).

The absolute difference between the ROC areas was 0.355 ± 0.0557 (95% CI: 0.245–0.464), with a z statistic of 6.368 and *p* < 0.0001, indicating a superiority for changes in NETest2.0^®^ scores versus changes in CgA for identifying progression (Table 2).

### 3.4. Primary Monitoring Analysis

**Figure 4:**
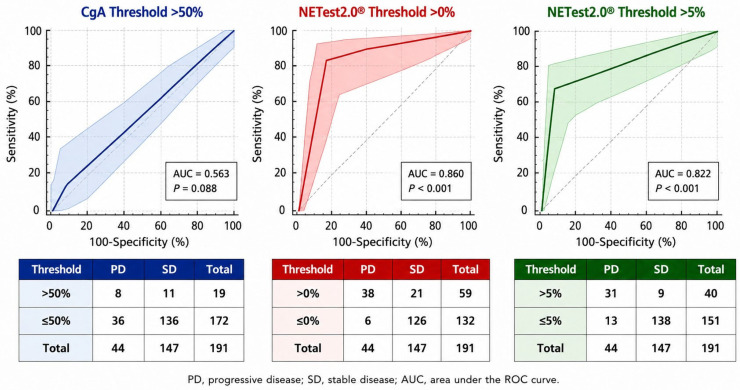
Head-to-head comparisons for CgA threshold vs. two different NETest thresholds using 3-point AUC analysis; 2 × 2 tables for each of the approaches/thresholds are included below. The dotted lines reflect the 95% confidence interval.

When these thresholds were applied to the primary monitoring objective (using the CASPAR protocol), CgA showed a limited discrimination for detecting progression. The assay results had an AUC of 0.553 ± 0.0314 (95% CI: 0.480–0.625) (Figure 4, Table 2).

ROC analysis evaluating NETest2.0^®^ delta thresholds demonstrated an AUC of 0.860 ± 0.0299 (95% CI: 0.803–0.906), whereas a threshold of >5% for the assay yielded an AUC of 0.822 ± 0.0362 (95% CI: 0.760–0.873).

Both thresholds significantly outperformed the CgA-based approach. The difference in AUC between CgA and NETest2.0^®^ (T > 0%) was 0.307 ± 0.0459 (95% CI: 0.217–0.397; z = 6.684; *p* < 0.0001). The corresponding difference between CgA and NETest2.0^®^ (T > 5%) was 0.268 ± 0.0468 (95% CI: 0.176–0.360; z = 5.732; *p* < 0.0001).

### 3.5. Operating Characteristics Across Thresholds

At the conventional CgA surveillance threshold (TΔ: >50%), sensitivity was only 18.18%, despite a specificity of 92.52% and an overall accuracy of 75.39% (Table 3).

In comparison, NETest2.0^®^ (TΔ: >0%) exhibited a sensitivity of 86.36% with a specificity of 85.71% and an accuracy of 85.86%. NETest2.0^®^ (TΔ: >5%) yielded the best overall balance of discrimination, with a sensitivity of 70.45%, a specificity 93.88%, a PPV 77.50%, an NPV 91.39%, and an accuracy 88.48%.

### 3.6. Multivariable and Logistic Regression Analyses

**Figure 5:**
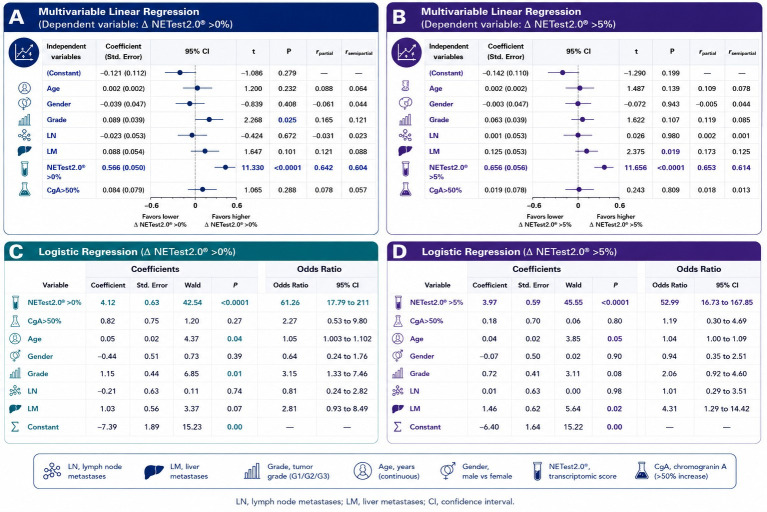
MVA and LRA of clinical data, the CgA threshold and the two different NETest2.0 thresholds. (**A**,**B**) Multivariable regression analyses using either TΔ: >0% (**A**) or TΔ: >5% (**B**). (**C**,**D**) Logistic regression analyses demonstrating the association between clinicopathologic variables and progression outcomes using TΔ: >0% (**C**) and TΔ: >5% (**D**) NETest2.0^®^ thresholds.

MVA were performed independently using NETest2.0^®^ thresholds of TΔ: >0% and TΔ: >5% to identify variables associated with disease progression (Figure 5A,B). Across both models, changes in NETest2.0^®^ scores emerged as the strongest independent predictor of progression (both *p* < 0.0001), demonstrating the highest statistical contribution to the model (t = 11.33–11.66).

In the TΔ: >0% model, tumor grade was additionally identified as an independent predictor of progression, although with substantially lower effect strength compared with NETest2.0^®^ dynamics (t = 2.27, *p* = 0.025). In contrast, in the TΔ: >5% model, grade was no longer retained as a significant variable, whereas liver metastases became independently associated with progression (t = 2.37, *p* = 0.019). Importantly, CgA TΔ: >50% was not identified as a significant contributor in either model.

LRA confirmed that changes in NETest2.0^®^ represented the dominant factor associated with progression outcomes (both *p* < 0.0001; Figure 5C,D). NETest2.0^®^ demonstrated markedly elevated odds ratios ranging from 52.99 to 61.26, substantially exceeding those of other clinicopathologic variables. Additional factors associated with progression included age (OR: 1.04–1.05 per year increase), tumor grade (OR: 2.06–3.15), and liver metastases (OR: 2.91–4.31), depending on the model used. Consistent with the MVA findings, CgA >50% did not significantly contribute to progression prediction.

Of note is that patients with liver metastases had significantly higher baseline NETest2.0^®^ scores for those with liver metastases (66.7 ± 16.5) versus no liver metastases (60.8 ± 17.3, *p* = 0.007) while grade was also associated with baseline scores (G1: 59.8 ± 16.8; G2: 66.3 ± 15.9 and G3: 69.6 ± 14,7; Kruskal–Wallis test: *p* = 0.0063). This identifies that higher baseline NETest scores are associated with both higher tumor grade and the presence of liver metastases. It is clear, however, that neither grade nor liver metastasis necessarily predicts the change in NETest over time, which supports the interpretation that longitudinal ΔNETest reflects dynamic changes in disease activity rather than simply baseline disease severity.

### 3.7. Sensitivity Analysis of Sampling Interval Effects

A sensitivity analysis was performed to determine whether sampling interval/PFS influences ΔNETest magnitude or progression classification. Firstly, PFS was not associated with signed ΔNETest (Pearson r = 0.059, *p* = 0.419; Spearman rho = 0.108, *p* = 0.138). Secondly, PFS showed only a weak Pearson association with |ΔNETest| (r = −0.143, *p* = 0.048), which was not confirmed by Spearman analysis (rho = −0.100, *p* = 0.169), bootstrap inference, quartile analyses, or outlier sensitivity analyses. Finally, PFS was not an independent predictor of progression status in logistic regression (OR per month = 1.037, *p* = 0.154).

## 4. Discussion

This registry-based sub-study was designed to evaluate clinical validity and comparative biomarker performance rather than prospective clinical utility or management outcomes. It demonstrated that NETest2.0^®^ provides materially better monitoring performance than CgA in patients with NET. Across both exploratory and primary analyses, serial NETest2.0^®^ change discriminated progressive from non-progressive disease with substantially higher AUCs than CgA, and the magnitude of the effect was statistically robust, with all key comparisons yielding *p* < 0.0001.

The issue of monitoring is particularly relevant in NET because radiographic progression may evolve slowly, lesions are often difficult to quantify reproducibly, and treatment decisions frequently depend on subtle longitudinal changes rather than rapid volumetric growth [9]. Consequently, inaccurate biomarkers may either delay recognition of progression or trigger unnecessary imaging and intervention [25].

One of the most striking observations was the near-chance performance of CgA in the exploratory analysis (based on changes between first and second samples). This biomarker also exhibited a poor performance in the standardized, predefined surveillance framework. Although CgA remains embedded in many clinical pathways, its biological limitations are substantial [9,13,15]. CgA reflects secretion from neurosecretory granules rather than tumor growth directly, and expression varies according to tumor differentiation, secretory phenotype, renal clearance, proton-pump inhibitor exposure, inflammation, and assay platform variability [26,27]. In addition, there are several kits that may be used, each with different sensitivities, specificities and overall accuracies [28]. Of note is that the >50% of samples (at both time points) were CgA-negative. These confounding influences may explain the poor dynamic relationship between serial CgA measurements and imaging-defined progression observed in the present study. They also confirm the need for an accurate biomarker for NET detection.

By contrast, NETest2.0^®^ was positive in the majority of cases and changes in scores captured progression-related molecular change with high discriminatory power [19]. A highly sensitive strategy was achieved using any upward NETest2.0^®^ drift (>0%), whereas a modestly more conservative threshold (>5%) improved specificity and overall accuracy. The >5% threshold should be interpreted as a specificity-enhancing threshold that reduces the impact of small fluctuations around the baseline. In contrast, the >0% threshold prioritizes sensitivity and may capture an early biological signal but is expected to be more susceptible to minor analytical or biological variation. This flexibility reflects the expected trade-off between sensitivity and specificity across predefined operating thresholds. In higher-risk settings, sensitivity may be prioritized, whereas in lower-risk or imaging-sparing settings, a slightly higher threshold may be preferred to reduce false-positive prompts for further assessment. The >0% threshold prioritizes sensitivity by capturing any upward molecular drift, but small changes around zero may include both early biological signal and minor analytical or biological fluctuation. The >5% threshold was therefore evaluated as a more conservative operating point designed to reduce the influence of low-amplitude noise while preserving clinically useful discrimination [29].

The biological rationale for the superior performance of NETest2.0^®^ is plausible. In contrast to CgA, NETest2.0^®^ integrates multiple biologic domains relevant to NET behavior, including proliferation signaling, metabolic regulation, neuroendocrine secretion pathways, receptor signaling, epigenetic modulation, and tumor-associated cellular activation [30]. This systems-biology approach explains the superior capacity of the assay to detect dynamic biologic changes preceding or accompanying radiographic progression. This approach is consistent with the evolving role of liquid biopsy in oncology, where composite molecular signatures are increasingly recognized as more informative than single-analyte biomarkers [9].

While the present study is enriched for GEP-NETs, the assay interrogates a multigene neuroendocrine transcriptomic program rather than a biomarker restricted to a single anatomic site. The findings should therefore be interpreted as strongest for the population studied, while supporting further prospective evaluation across bronchopulmonary NETs, unknown primary NETs, and other neuroendocrine neoplasms.

These data support two clinically relevant interpretations. First, the most sensitive NETest2.0^®^ monitoring approach was obtained with any increase above the baseline (>0%). Secondly, a modestly higher threshold (>5%) traded some sensitivity for greater specificity while maintaining a clear overall advantage relative to CgA. The observation that even minimal increases in NETest2.0^®^ (>0%) retained high sensitivity suggests that molecular transcriptomic drift will likely precede overt radiographic progression. Conversely, the application of a slightly higher threshold (>5%) reduced any biologic or assay-based noise and improved specificity while maintaining robust discriminatory power. These findings raise the possibility that NETest2.0^®^ thresholds could be individualized according to clinical context, disease burden, or surveillance intensity [19]. This is an active area of investigation. The observed reduction in NETest2.0 positivity between sequential samples should be interpreted in the context of surveillance and treatment monitoring. In patients with stable disease, decreasing or low transcriptomic activity may reflect treatment effect, tumor quiescence, or suppression of biologic activity despite the persistence of structural disease on imaging.

The data also support a practical role for NETest2.0^®^ as a biology-guided adjunct to imaging rather than a replacement for imaging. In long-term follow-up, particularly after surgery or during treatment monitoring, NETest2.0^®^ could enrich surveillance by identifying patients who merit closer radiological assessment while potentially reducing unnecessary imaging in those with persistently low-risk molecular profiles. Importantly, NETest2.0^®^ changes consistently outperformed conventional clinicopathologic variables in both multivariable and logistic regression models. The very high odds ratios associated with transcriptomic progression suggest that molecular activity captured by NETest2.0^®^ represents a more direct reflection of active tumor biology than static anatomical descriptors such as grade or metastatic distribution.

We also evaluated whether the sampling interval had any impact on changes in the NETest2.0 score of PFS. The sensitivity analyses confirmed that sampling interval/PFS did not meaningfully influence signed ΔNETest, absolute ΔNETest magnitude, or progression classification. The small Pearson association between PFS and |ΔNETest| observed in the primary analysis was not robust to non-parametric testing, outlier exclusion, stratified analyses, bootstrap inference, or leave-one-out assessment. Overall, these data support the conclusion that ΔNETest-based progression classification reflects biological disease activity rather than sampling interval artifact.

This study does have some limitations. The analysis was registry-based, the imaging comparator was determined by routine clinical practice rather than by a single protocol, and the present manuscript reformats the statistical outputs contained in the source document rather than performing an independent reanalysis of the raw patient-level dataset. The cohort also included diverse clinical scenarios, treatment strategies, and disease stages, which may introduce biologic variability. In addition, relatively few G3 tumors were included, limiting subgroup inference for higher-grade disease. Different CgA assay platforms were used according to clinical site practice. This reflects real-world surveillance conditions but may have contributed to the weak performance of CgA because CgA assays differ in calibration, analytical sensitivity, and reference ranges. Nevertheless, the internal consistency of the ROC and operating characteristic outputs supports the central conclusion that serial NETest2.0^®^ monitoring outperforms CgA in this cohort. These data also further support the notion that monitoring using CgA is problematic. Importantly, discordance between molecular and imaging assessments should not necessarily be interpreted as assay failure. Molecular progression may precede radiographic progression, particularly in low-volume disease or during transitional biologic states [31]. Further datapoints would be helpful to determine if molecular suppression during therapy may transiently reduce circulating transcriptomic activity despite persistent structural abnormalities on imaging [32].

## 5. Conclusions

Serial NETest2.0^®^ assessments demonstrated markedly superior performance compared with CgA for identifying NET progression in a real-world surveillance cohort. The findings support the concept that transcriptomic liquid biopsy more accurately reflects dynamic tumor biology than conventional monoanalyte biomarkers. The incorporation of NETest2.0^®^ into longitudinal surveillance algorithms may improve biologically guided monitoring, optimize imaging utilization, and facilitate earlier recognition of clinically meaningful progression.

## Figures and Tables

**Table 1:** Study design and analytic framework.

Characteristic	Value
Registry/design	RegisterNET (NCT02270567); registry-based surveillance study
Cohort	191 patients with paired blood samples *
Sampling	Two serial blood draws; imaging performed at second timepoint (blood sample #2) **
Reference standard	Imaging and clinical adjudication (RECIST 1.1 where applicable)
Index/comparator	NETest2.0^®^ versus serum Chromogranin A ***
NETest2.0^®^ threshold	Score ≥ 50 at second timepoint (imaging)
Thresholds	NETest2.0^®^ > 0%, >5% versus CgA > 50%
Primary endpoint	Progressive versus non-progressive disease

* A total of 147 were stable at follow-up, whole 44 showed evidence of progressive disease, ** median follow-up 8 months (range: 1–36; IQR: 3–14), *** CgA platform was not standardized.

**Table 2:** ROC performance of CgA and NETest2.0^®^ monitoring strategies.

Analysis	AUC	SE	95% CI	Δ AUC vs. CgA (95% CI)	*p* Value
ΔCgA (exploratory) *	0.538	0.0503	0.465–0.610	Reference	—
ΔNETest2.0^®^ (exploratory) *	0.893	0.0249	0.840–0.933	0.355 (0.245–0.464)	<0.0001
CgA T > 50% (primary) **	0.553	0.0314	0.480–0.625	Reference	—
NETest2.0^®^ T > 0% **	0.860	0.0299	0.803–0.906	0.307 (0.217 to 0.397)	<0.0001
NETest2.0^®^ T > 5% **	0.822	0.0362	0.760–0.873	0.268 (0.176 to 0.360)	<0.0001

* Based on changes in measurements of the 2 samples. ** Based on the threshold evaluation AUC = area under the curve, CgA = Chromogranin A, CI = confidence interval, SE = standard error, T = threshold evaluated.

**Table 3:** Operating characteristics of CgA and NETest2.0^®^ thresholds.

Threshold	Sensitivity (%)	Specificity (%)	PLR	NLR	PPV (%)	NPV (%)	Accuracy (%)
CgA TΔ: >50%	18.18	92.52	2.43	0.88	42.11	79.07	75.39
NETest2.0^®^ TΔ: >0%	86.36	85.71	6.05	0.16	64.41	95.45	85.86
NETest2.0^®^ TΔ: >5%	70.45	93.88	11.51	0.31	77.50	91.39	88.48

## Data Availability

Due to privacy and ethical concerns, the data that support the findings of this study are not publicly available but are available on request from the corresponding author.

## Supplementary Materials

The following supporting information can be downloaded at: https://www.mdpi.com/article/10.3390/cancers18142206/s1, Table S1: STROBE Checklist.

## Abbreviations

The following abbreviations are used in this manuscript:

AUC	Area under the curve
cDNA	Complementary DNA
CI	Confidence interval
CgA	Chromogranin A
CT	Computed Tomography
ENETS	European Neuroendocrine Tumor Society
GEP	Gastroenteropancreatic
LRA	Logistic regression analysis
mRNA	Messenger RNA
MRI	Magnetic Resonance Imaging
MVA	Multivariable analysis
OR	Odds ratio
NEN	Neuroendocrine neoplasia
NET	Neuroendocrine tumor
NLR	Negative likelihood ratio
NPV	Negative predictive value
PCR	Polymerase Chain Reaction
PD	Progressive disease
PET	Positron emission tomography
PLR	Positive likelihood ratio
PPV	Positive predictive value
RECIST	Response Evaluation Criteria in Solid Tumors
RNA	Ribonucleic acid
ROC	Receiver operating characteristic
SD	Stable disease
TNM	Tumor, Node, and Metastasis
