# Supplementary material for: NETest2.0® Demonstrates Superior Monitoring Performance Compared with Chromogranin A in Neuroendocrine Tumor Surveillance
Source: Cancers (Basel). 2026 Jul 9;18(14):2206. doi: 10.3390/cancers18142206 (PMC13406635; doi:10.3390/cancers18142206)
Supplement: Supplementary file 1 [file cancers-18-02206-s001.zip › cancers-4383613-supplementary.pdf]

cancers

Supplementary Materials

NETest2.0® Demonstrates Superior Monitoring Performance Compared with Chromogranin A in Neuroendocrine Tumor Surveillance

Kiarash Mashayekhi [1], Mark Kidd [2,*] and Anthony Gulati [3]

[1] Department of Surgery, University of North Dakota School of Medicine & Health Sciences, Grand Forks, ND 58203, USA; kia.mashayek@alumni.usc.edu
[2] Wren Laboratories, Branford, CT 06405, USA
[3] Department of Medical Oncology, Stamford Health, Stamford, CT 06902, USA; agulati@stamhealth.org
* Correspondence: mkidd@wrenlaboratories.com; Tel.: +1-203-208-3464

STROBE Checklist

Manuscript: Clinical Validity of NETest2.0® in Surveillance of Neuroendocrine Tumor Patients: Evidence from a NET Registry Study (NCT02270567)

Study Design: Prospective observational registry-based cohort study

Table S1. STROBE Checklist.

STROBE Item	Recommendation	Manuscript Location
1a	Indicate study design in title/abstract	Title, Abstract
1b	Informative abstract	Abstract
2	Scientific background and rationale	Introduction
3	Objectives and hypotheses	Introduction
4	Present key elements of study design early	Methods
5	Describe setting, locations, dates	Methods—RegisterNET Registry
6a	Eligibility criteria and participant selection	Methods—Study Population
6b	Matching criteria (if applicable)	Not applicable
7	Define outcomes, exposures and predictors	Methods—Endpoints
8	Data sources and measurement methods	Methods—Biomarker Testing
9	Describe efforts to address bias	Methods—Statistical Analysis
10	Explain study size	Methods—Study Population
11	Explain quantitative variables	Methods—Statistical Analysis
12a	Statistical methods	Methods—Statistical Analysis
12b	Methods for subgroup analyses	Methods—Statistical Analysis
12c	Missing data handling	Methods—Statistical Analysis
12d	Loss to follow-up	Registry follow-up described in Methods
12e	Sensitivity analyses	Results
13a	Participant numbers at each stage	Results; Figure 1
13b	Reasons for exclusion	Figure 1
13c	Flow diagram	Figure 1
14a	Participant characteristics	Table 1
14b	Missing data	Results

STROBE Item	Recommendation	Manuscript Location
14c	Follow-up time	Results
15	Outcome events or summary measures	Results
16a	Main results with estimates	Results; Tables 2–4
16b	Category boundaries	Methods
16c	Translation of relative to absolute risk	Not applicable
17	Other analyses	Results
18	Key findings	Discussion
19	Limitations	Discussion
20	Interpretation	Discussion
21	Generalizability	Discussion
22	Funding	Funding Statement

The authors confirm adherence to STROBE reporting recommendations for observational cohort studies.
